# High‐Efficiency Polymer:Nonfullerene Solar Cells with Quaterthiophene‐Containing Polyimide Interlayers

**DOI:** 10.1002/advs.201800331

**Published:** 2018-06-10

**Authors:** Euyoung Park, Jooyeok Seo, Hyemi Han, Hwajeong Kim, Youngkyoo Kim

**Affiliations:** ^1^ Organic Nanoelectronics Laboratory KNU Institute for Nanophotonics Applications (KINPA) Department of Chemical Engineering School of Applied Chemical Engineering Kyungpook National University Daegu 41566 Republic of Korea; ^2^ Priority Research Center Research Institute of Advanced Energy Technology Kyungpook National University Daegu 41566 Republic of Korea

**Keywords:** interfacial layers, nonfullerene acceptors, organic solar cells, polyimides, stability

## Abstract

Interfacial layers (interlayers) are one of the emerging approaches in organic solar cells with bulk heterojunction (BHJ) layers because the solar cell efficiency can be additionally improved by their presence. However, less attention is paid to the use of interlayers for polymer:nonfullerene solar cells, which have strong advantages over polymer:fullerene solar cells. In addition, most polymers used for the interlayers possess a low glass transition temperature (*T*_g_). Here, it is demonstrated that two types of quarterthiophene‐containing polyimides (PIs) with high *T*_g_ (>198 °C), which are synthesized using pyromellitic dianhydride (PMDA) and cyclobutane‐1,2,3,4‐tetracarboxylic dianhydride (CTCDA), can act as an interfacial layer in the polymer:nonfullerene solar cells with the BHJ layers of poly[(2,6‐(4,8‐bis(5‐(2‐ethylhexyl)thiophen‐2‐yl)‐benzo[1,2‐*b*:4,5‐*b*′]dithiophene))‐*alt*‐(5,5‐(1′,3′‐di‐2‐thienyl‐5′,7′‐bis(2‐ethylhexyl)benzo[1′,2′‐c:4′,5′‐c′]dithiophene‐4,8‐dione))] (PBDB‐T) and 3,9‐bis(2‐methylene(3‐(1,1‐dicyanomethylene)‐indanone))‐5,5,11,11‐tetrakis(4‐hexylphenyl)‐dithieno[2,3‐d:2′,3′‐d′]‐*s*‐indaceno[1,2‐b:5,6‐b′]dithiophene) (ITIC), or (3‐(1,1‐dicyanomethylene)‐1‐methyl‐indanone)‐5,5,11,11‐tetrakis(4‐hexylphenyl)‐dithieno[2,3‐d:2′,3′‐d′]‐*s*‐indaceno[1,2‐b:5,6‐b′]‐dithiophene) (IT‐M). Interestingly, the efficiency and stability of devices are improved by the PMDA‐based PI interlayers with a stretched chain structure but degraded by the CTCDA‐based PI interlayers with a bended chain structure.

## 1 Introduction

Interfacial layers (or interlayers) have attracted keen attention in organic solar cells because they enable efficient charge transfer from light‐absorbing active layers to corresponding charge‐collecting buffer layers.^1^, ^2^, ^3^, ^4^, ^5^ To date, most interfacial layers have been made of polymers and extensively applied for organic solar cells with bulk heterojunction (BHJ) films of electron‐donating (p‐type) conjugated polymers and electron‐accepting (n‐type) fullerene derivatives, so‐called “polymer:fullerene” solar cells.^6^, ^7^, ^8^, ^9^, ^10^, ^11^, ^12^, ^13^, ^14^ Recently, the power conversion efficiency (PCE) of “polymer:nonfullerene” solar cells with nonfullerene‐type electron acceptors has reached ≈11–13% by introducing polymer donors with bithienylbenzodithiophene (BDTT)‐based units and nonfullerene acceptors with indacenodithienothiophene (IT)‐based derivatives.^15^, ^16^, ^17^, ^18^, ^19^ However, less attention has been paid to applying such interfacial layers for polymer:nonfullerene solar cells, particularly for the inverted‐type device structures that benefit from the use of stable top electrodes with high work functions including, e.g., silver (see Table S1 in the Supporting Information).^20^, ^21^, ^22^, ^23^, ^24^, ^25^, ^26^, ^27^, ^28^, ^29^, ^30^

Basically, the polymeric interfacial layers can be classified into two different types, insulating polymers and ionic polymers, according to the molecular structure. Both insulating and ionic polymers induce a dipole layer on charge‐collecting buffer layers (mostly metal oxides), leading to the work function shift in the metal oxide buffer layers, which eventually results in the increased built‐in potential inside devices.^31^, ^32^, ^33^, ^34^ The built‐in potential increase can enhance the charge transport inside solar cells, which delivers the enhanced PCE. The thickness of the insulating polymer interlayers is typically limited to several nanometers or less because of their high electrical resistances, whereas ten times thicker interlayers can be allowed for the ionic polymer interlayers.^35^

Taking into account the ultrathin states at a scale of several nanometers or less, the insulating polymers for interlayers should be thermally and physicochemically stable for the long‐term durability of organic solar cells. However, conventional insulating polymers used for interlayers have low thermal stability, for example, as noted from their glass transition temperature (*T*_g_), of *T*_g_ = −28.15 °C for polyethyleneimine (PEI), *T*_g_ = −24 °C for polyethyleneimine‐ethoxylated (PEIE), and *T*_g_ = 54 °C for poly(2‐ethyl‐2‐oxazoline) (PEOz).^36^, ^37^, ^38^, ^39^ Therefore, it is strongly required to develop highly stable polymers for the insulating polymer interlayers toward durable organic solar cells.

In this work, we have synthesized quarterthiophene (QT)‐based polyimides (PIs) and applied them as a stable interlayer for polymer:nonfullerene solar cells. Two different dianhydrides, pyromellitic dianhydride (PMDA) and cyclobutane‐1,2,3,4‐tetracarboxylic dianhydride (CTCDA), were employed to understand the influence of molecular structure on the performance of solar cells. The PI interlayers were formed by thermal imidization of their precursor poly(amic acid) (PAA) films coated on the electron‐collecting buffer layers (zinc oxide—ZnO) in the inverted‐type polymer:nonfullerene solar cells, of which BHJ layers consist of poly[(2,6‐(4,8‐bis(5‐(2‐ethylhexyl)thiophen‐2‐yl)‐benzo[1,2‐b:4,5‐b′]dithiophene))‐*alt*‐(5,5‐(1′,3′‐di‐2‐thienyl‐5′,7′‐bis(2‐ethylhexyl)benzo[1′,2′‐c:4′,5′‐c′]dithiophene‐4,8‐dione))] (PBDB‐T) and 3,9‐bis(2‐methylene(3‐(1,1‐dicyanomethylene)‐indanone))‐5,5,11,11‐tetrakis(4‐hexylphenyl)‐dithieno[2,3‐d:2′,3′‐d′]‐s‐indaceno[1,2‐b:5,6‐b′]dithiophene) (ITIC), or (3‐(1,1‐dicyanomethylene)‐1‐methyl‐indanone)‐5,5,11,11‐tetrakis(4‐hexylphenyl)‐dithieno[2,3‐d:2′,3′‐d′]‐s‐indaceno[1,2‐b:5,6‐b′]‐dithiophene) (IT‐M). In order to confirm the influence of the PI interlayers, inverted‐type polymer:fullerene solar cells were fabricated by employing poly[4,8‐bis(5‐(2‐ethylhexyl)thiophen‐2‐yl)benzo[1,2‐b:4,5‐b]dithiophene‐*alt*‐3‐fluorothieno[3,4‐b]thiophene‐2‐carboxylate] (PTB7‐Th) and the [6,6]‐phenyl‐C_71_‐butyric acid methyl ester (PC_71_BM) for the BHJ layers.

## 2 Results and Discussion

**Figure 1:**
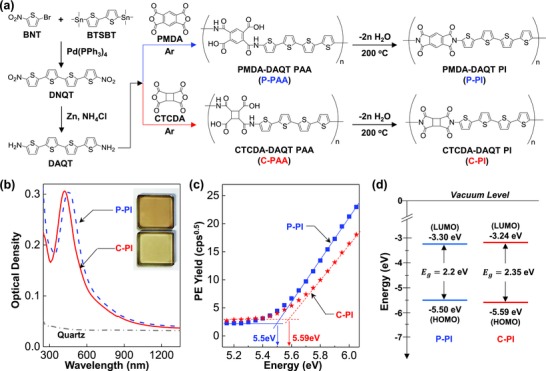
Synthesis, optical absorption, and energy band diagram. a) Scheme for the synthesis of DAQT, precursor poly(amic acid)s (P‐PAA and C‐PAA), and polyimides (P‐PI and C‐PI). b) Normalized optical absorption spectra for the P‐PI and C‐PI films coated on quartz substrates (see the inset photographs). c) Photoelectron (PE) yield spectra for the P‐PI and C‐PI films (see onset points for each spectrum). d) Flat energy band diagram for the P‐PI and C‐PI films: *E*_g_ denotes optical bandgap energy.

First, as shown in **Figure** **1**a, 5,5″‐diamino‐2,2′:5′,2″:5″,2′″‐quaterthiophene (DAQT) was obtained via reduction reaction of 5,5′″‐dinitro‐2,2′:5′,2″:5″,2′″‐quaterthiophene (DNQT) that was synthesized by the Stille coupling between 2‐bromo‐5‐nitrothiophene (BNT) and 5,5′‐bis(trimethylstannyl)‐2,2′‐bithiophene (BTSBT) (see details in the “Methods for Synthesis and Characterization” section and Figures S1–S3 in the Supporting Information). Next, DAQT was reacted with the two different dianhydrides, PMDA and CTCDA, leading to corresponding PAA, poly(2‐((5′″‐methyl‐[2,2′:5′,2″:5″,2′″‐quaterthiophen]‐5‐yl)carbamoyl)‐5‐(methylcarbamoyl)terephthalic acid) (PMDA–DAQT PAA) and poly(2‐((5′″‐methyl‐[2,2′:5′,2″:5″,2′″‐quaterthiophen]‐5‐yl)carbamoyl)‐4‐(methylcarbamoyl)cyclobutane‐1,3‐dicarboxylic acid) (CTCDA–DAQT PAA). These PAA precursor polymers were soluble in a weak base solvent such as dimethylformamide (DMF) and *N*,*N*‐dimethylacetamide (DMAc) so that their solutions could be used for the spin‐coating of corresponding films on substrates. Then, the coated PAA films were converted to corresponding PI films, PMDA–DAQT PI (P‐PI in short) and CTCDA–DAQT PI (C‐PI in short), via a thermal imidization process at 200 °C for 90 min. The conversion from PAA to PI was confirmed by the Fourier transform‐infrared (FT‐IR) spectroscopy measurements: The PI films showed new peaks such as C=O asymmetric (1856 cm^−1^) and symmetric (1787 and 1790 cm^−1^) stretching, C—N stretching (1380 and 1376 cm^−1^), and C=O bending (716 and 740 cm^−1^) of imide rings, whereas the peaks in the PAA films such as C=O stretching (1730 and 1723 cm^−1^) of carboxylic acid groups and N—H bending (1600 and 1560 cm^−1^) of amide groups were pronouncedly reduced and/or disappeared (see Figure S5 in the Supporting Information).^40^, ^41^, ^42^ The *T*_g_ of the P‐PI and C‐PI samples, which were imidized at the same condition (200 °C for 90 min), reached ≈198 and 219 °C, respectively (see Figure S6 in the Supporting Information). The *T*_g_ onset was further increased up to >220 °C in the case of thermal imidization at 250 °C for 90 min. Here it is noted that the *T*_g_ of thin films (interlayers) can be further reduced from the values measured for bulk (powder) samples as reported in previous studies.^41^, ^43^, ^44^, ^45^

As shown in Figure 1b, the main optical absorption peak was measured at 452 nm for P‐PI and 419 nm for C‐PI in the presence of long absorption tails over 1200 nm, which can be attributable to the formation of exciplex by the chain stacking as typically observed in PIs.^46^, ^47^ The optical bandgap energy (*E*_g_) was obtained 2.2 eV for P‐PI and 2.35 eV for C‐PI from the Tauc plots (see Figure S7 in the Supporting Information). The highest occupied molecular orbital (HOMO) energy, which was extracted from the onset point of photoelectron (PE) yield spectra, was 5.5 eV for P‐PI and 5.59 eV for C‐PI (see Figure 1c). From the *E*_g_ and HOMO energy values, the lowest unoccupied molecular orbital (LUMO) energy was calculated to 3.30 eV for P‐PI and 3.24 eV for C‐PI (see Figure 1d).

**Figure 2:**
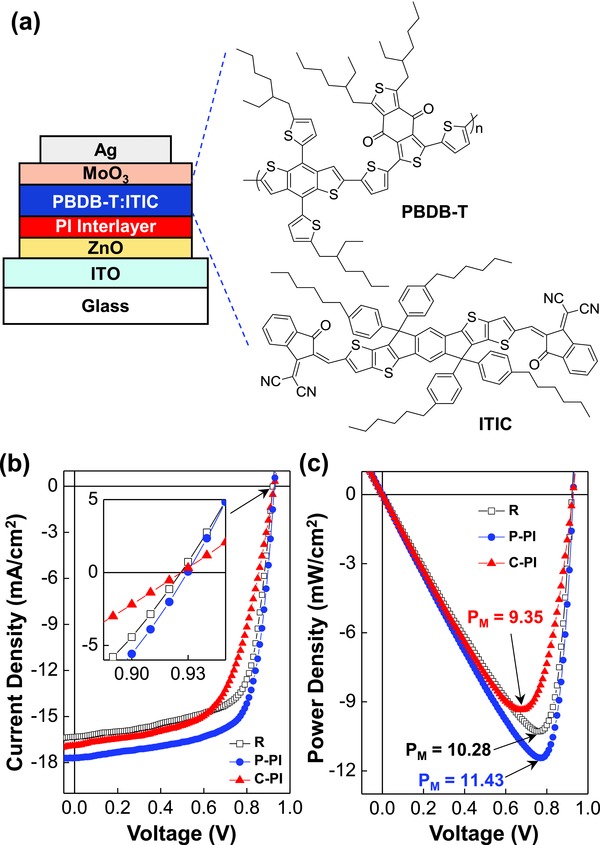
Device structure and performances. a) Illustration of device cross section and materials used for the inverted‐type PBDB‐T:ITIC solar cells. b) Light (air mass 1.5G, 100 mW cm^−2^) *J*–*V* curves for the PBDB‐T:ITIC solar cells without (R) and with the PI interlayers (P‐PI and C‐PI): Inset shows the enlarged *J*–*V* curves focusing on the open‐circuit region. c) Power density–voltage curves calculated from the *J*–*V* curves in panel (b). The maximum power density is given on each curve.

Next, both P‐PI and C‐PI were applied as an interfacial layer for inverted‐type polymer:nonfullerene solar cells with the PBDB‐T:ITIC BHJ layers (see **Figure** **2**a). The current density–voltage (*J*–*V*) curves in Figure 2b unveiled that the performance of solar cells was pronouncedly improved by applying the P‐PI interlayer (≈2 nm thick). However, when the C‐PI interlayer was introduced, the current density in the *J*–*V* curve was noticeably lowered at the voltages higher than 0.6 V even though the short‐circuit current density (*J*_SC_) was slightly increased. Consequently, as shown in Figure 2c, the maximum power density was improved by ≈10% (from 10.28 to 11.43 mW cm^−2^) for the P‐PI interlayer, whereas it was reduced by ≈9% (from 10.28 to 9.35 mW cm^−2^) for the C‐PI interlayer.

**Figure 3:**
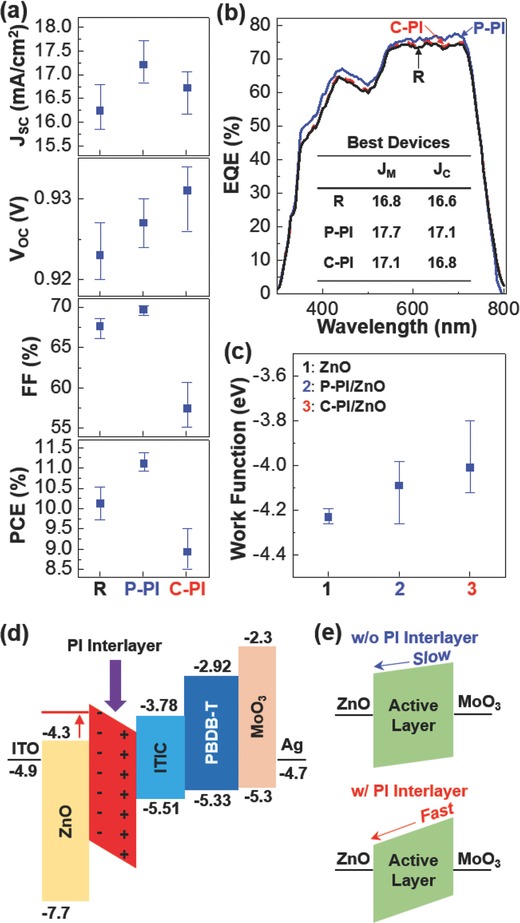
Solar cell parameters and work function change. a) Change of solar cell parameters for the PBDB‐T:ITIC solar cells without (R) and with the PI interlayers (P‐PI and C‐PI). b) EQE spectra for the PBDB‐T:ITIC solar cells without (R) and with the PI interlayers (P‐PI and C‐PI): *J*_M_ and *J*_C_ represent the measured short‐circuit current density from the *J*–*V* curves and the calculated current density integrated from the EQE spectra, respectively. c) Change of the ZnO work functions: 1) ZnO, 2) P‐PI on ZnO, and 3) C‐PI on ZnO. d) Illustration for the dipole formation by the presence of the PI interlayers in the flat energy band diagram of the present device: Note that the energy (eV) unit is emitted for simplicity. e) Illustration for the charge transport inside solar cells according to the presence of PI interlayers.

Further analysis on solar cell parameters, which were obtained from 30 devices, disclosed that both P‐PI and C‐PI interlayers increased *J*_SC_ in the PBDB‐T:ITIC solar cells but the P‐PI interlayer delivered better *J*_SC_ than the C‐PI interlayer (see **Figure** **3**a). Here the increased *J*_SC_ by the PI interlayers can be ascribed to the improved charge transport inside devices, as supported by the trend of electron mobility in Figure S8 (Supporting Information), because the external quantum efficiency (EQE) value was slightly improved without change of its shape (see Figure 3b). The open‐circuit voltage (*V*_OC_) of devices was also marginally increased by the presence of the two PI interlayers. In particular, the C‐PI interlayers delivered slightly higher *V*_OC_ than the P‐PI interlayers, which is supported by the lowered ZnO work function (see Figure 3c). In more detail, as depicted in Figure 3c, the formation of a dipole layer by the presence of the PI interlayers could increase the work function of the ZnO layers, which caused the built‐in potential increase leading to the faster electron transport inside devices (see Figure 3d,e). Here the fill factor (FF) of devices was also improved by the P‐PI interlayer, but it was remarkably decreased by the C‐PI interlayer. The reduced FF by the C‐PI interlayer can be apparently attributed to the increased series resistance (*R*_S_) from *R*_S_ = 0.11 kΩ cm^2^ (reference device) to *R*_S_ = 0.21 kΩ cm^2^ (C‐PI device), compared to *R*_S_ = 0.08 kΩ cm^2^ (P‐PI device). The increased *R*_S_ by the presence of the C‐PI interlayer can be ascribed to the highest interfacial resistance (see *R*2 in Figure S9 in the Supporting Information), as measured with impedance spectroscopy, which might be caused by the less desirable morphology formed in the bottom region (toward the ZnO layer) of the BHJ (PBDB‐T:ITIC) layer owing to the presence of the C‐PI interlayer. As a result, the P‐PI interlayer could enhance the PCE of devices up to 11.4%, whereas the C‐PI interlayer, rather, reduced the PCE to 9.5% (see **Table** **1**).

**Table 1:** Summary of solar cell parameters for the inverted‐type polymer:nonfullerene solar cells with the BHJ layers of PBDB‐T and ITIC or IT‐M under illumination with a simulated solar light (air mass 1.5G, 100 mW cm^−2^)

Devices	Acceptors	Parameters				
		*V*_OC_ [mV]	*J*_SC_ [mA cm^−2^]	FF [%]	PCE [%]	
					Average	Best
R	ITIC	924 (±3)	16.3 (±0.48)	67.4 (±1.2)	10.2 (±0.40)	10.6
	IT‐M	930 (±10)	16.5 (±0.27)	70.7 (±1.6)	10.8 (±0.24)	11.0
P‐PI	ITIC	927 (±3)	17.3 (±0.44)	69.6 (±0.6)	11.2 (±0.23)	11.4
	IT‐M	940 (±10)	17.2 (±0.33)	73.7 (±0.5)	11.9 (±0.27)	12.2
C‐PI	ITIC	930 (±4)	16.6 (±0.45)	57.9 (±2.8)	8.94 (±0.51)	9.5
	IT‐M	930 (±10)	15.8 (±0.17)	70.4 (±1.4)	10.3 (±0.36)	10.7

**Figure 4:**
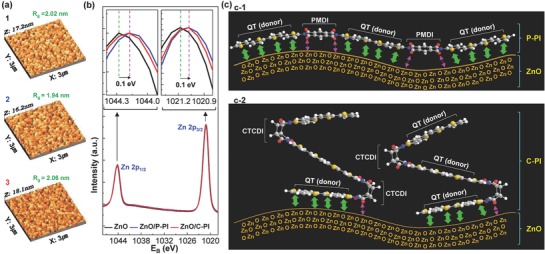
Surface morphology, XPS spectra, and energy‐minimized structure. a) 3D AFM images for the thin films coated on the ITO–glass substrates: 1) ZnO, 2) P‐PI on ZnO, and 3) C‐PI on ZnO. b) XPS spectra (Zn core level) for the thin films coated on the ITO–glass substrates. The two inset graphs denote the enlarged ones focusing on each peak. c) Illustration for the interaction between the energy‐minimized PI chains and the ZnO surfaces: c‐1) P‐PI has a stretched chain structure so as to maximize the interaction with the ZnO surface. c‐2) C‐PI has a bended chain structure leading to a limited interaction with the ZnO surface. The green bold arrows represent the interaction between the thiophene (donor) units in the PI chains and the oxygen (acceptor) components in the ZnO surface, while the pink thin arrows denote the interaction between the carbonyl dipoles in the PI chains and the Zn–O dipoles in the ZnO surface.

Here a question is arising on why the PCE of devices was improved by the P‐PI interlayer, not by the C‐PI interlayer, even though the ZnO work function was more shifted by the C‐PI interlayer than the P‐PI interlayer. To understand this, the surface state and molecular level interaction between ZnO and PI were investigated by employing atomic force microscopy (AFM), X‐ray photoelectron spectroscopy (XPS), and molecular simulation methods.^48^ As shown in **Figure** **4**a, the AFM measurement disclosed that the surface roughness (*R*_g_) was marginally changed after coating the PI interlayers due to the ultrathin feature. However, the phase angle (*Z*) was pronouncedly shifted from *Z* = 0.63° (bare ZnO layer) to *Z* = 1.97° (P‐PI) and *Z* = 1.82° (C‐PI) upon coating the PI interlayers (see the different phase images in Figure S10 in the Supporting Information). This result indicates that the PI interlayers are indeed present on the surface of the ZnO layers and make different surface states between the two PI interlayers. In addition, as shown in Figure 4b, the XPS measurement revealed that the Zn 2p peaks were shifted toward a lower energy region by the presence of the two PI interlayers but more spectral shift was measured for the P‐PI interlayers than the C‐PI interlayers even though the peak point shift was the same (Zn 2p_1/2_: from 1044.28 to 1044.18 eV and Zn 2p_3/2_: from 1021.18 to 1021.08 eV). This downshifted trend in the Zn 2p peaks reflects that the oxygen defects in the ZnO layers could be relatively reduced by the presence of the PI interlayers.^49^, ^50^, ^51^, ^52^ Therefore, it is shortly concluded that a special interaction was made between the ZnO surface and the PI interlayers. As observed from the energy minimized molecular structures in Figure 4c (see Figure S11 in the Supporting Information for 3D simulated molecular motions), the P‐PI chain has relatively a stretched shape so that it could make a good tight contact with the ZnO surface leading to a conformally coated surface. In contrast, the C‐PI chain has a bended shape so that a limited part in the C‐PI chain could directly contact the ZnO surface but the rest part should inevitably remain apart from the ZnO surface without direct contact (note that the cyclobutene groups in the CTCDI moieties are nonpolar so that they may have relatively low probability to make interactions with the polar ZnO surfaces). Therefore, the noncontact parts in the C‐PI chains might induce slightly increased electrical resistances, compared to the well‐contacted P‐PI chains, which can be supported by the relatively large FF drop for the devices with the C‐PI interlayers (see also the highest interfacial resistance in Figure S9 in the Supporting Information). Consequently, *J*_SC_ became lower for the C‐PI interlayers than the P‐PI interlayers.

**Figure 5:**
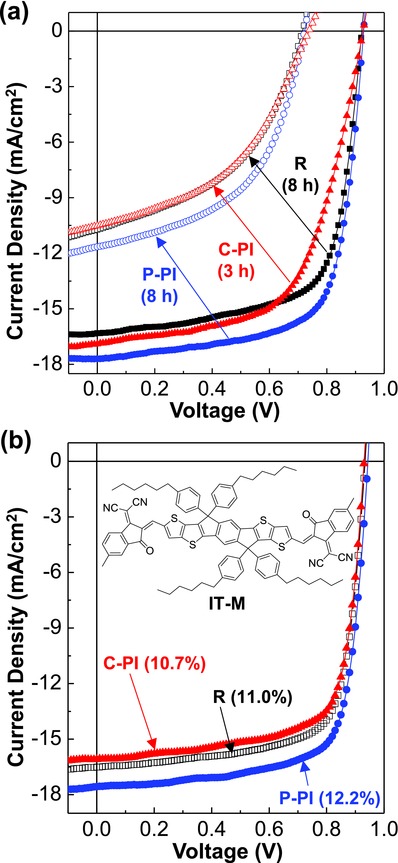
Stability and PBDB‐T:IT‐M solar cells. a) Change in the light (air mass 1.5G, 100 mW cm^−2^) *J*–*V* curves for the PBDB‐T:ITIC solar cells without (R) and with the PI interlayers (P‐PI and C‐PI). Note that the light *J*–*V* curves became poor after only 3 h of illumination in the case of the C‐PI interlayers, whereas the P‐PI interlayers delivered the best light *J*–*V* curves even after 8 h. b) Light (air mass 1.5G, 100 mW cm^−2^) *J*–*V* curves for the PBDB‐T:IT‐M solar cells without (R) and with the PI interlayers (P‐PI and C‐PI). The highest PCE of 12.2% was measured for the P‐PI interlayers.

Then the best devices with the PI interlayers were subjected to the brief lifetime test in order to investigate the influence of the P‐PI interlayers on the stability of devices. As shown in **Figure** **5**a, the light *J*–*V* curves of all devices fabricated were significantly degraded after 3–8 h of illumination with the simulated solar light (100 mW cm^−2^). This can be ascribed to the deterioration of active layers, as supported by the color change in the PBDB‐T:ITIC films. The devices with the C‐PI interlayers exhibited much quicker degradation even after 3 h, whereas the P‐PI interlayers delivered still better *J*–*V* curves than the reference devices after 8 h. This result reflects that the limited (partial) contact of the C‐PI chains with the ZnO surface (see the energy minimized molecular structures in Figure 4c) might generate unstable interfaces leading to the fast degradation in device performances. In contrast, the P‐PI interlayers might make stable interfaces with the ZnO layers so that they could have a positive influence on the device stability.

Finally, the P‐PI interlayers were applied for another polymer:nonfullerene solar cells with the PBDB‐T:IT‐M BHJ layers in order to confirm their effects. As shown in Figure 5b, the light *J*–*V* curve of the PBDB‐T:IT‐M solar cells was noticeably improved by the presence of the P‐PI interlayer, whereas the C‐PI interlayer degraded the light *J*–*V* curve. This result is in good agreement with the performance trend of the PBDB‐T:ITIC solar cells according to the type of PI interlayers. The maximum PCE of the PBDB‐T:IT‐M solar cells reproducibly reached 12.2% by introducing the P‐PI interlayers (see Table 1). It is also noteworthy that the performance of the inverted‐type PTB7‐Th:PC_71_BM solar cells could be improved by inserting the P‐PI interlayer even though it was rather lowered by the presence of the C‐PI interlayer (see Figure S13 in the Supporting Information).

## 3 Conclusion

In summary, quarterthiophene‐containing polyimides (P‐PI and C‐PI) with high *T*_g_ of >198 °C were synthesized and applied as an interlayer for the inverted‐type polymer:nonfullerene solar cells. The two PI interlayers (≈2 nm thick) were formed on the ZnO electron‐collecting buffer layers via thermal imidization of corresponding precoated PAA precursor films. The performances of polymer:nonfullerene solar cells were improved by the presence of the P‐PI interlayers, whereas the C‐PI interlayers degraded the device performances. The contrary effect of the two PI interlayers has been assigned to the different molecular structures. The P‐PI interlayers with a stretched chain structure could also improve the stability of solar cells, but the device performance was quickly degraded in the case of the C‐PI interlayers with a bended chain structure (note that the similar trend in device performance was measured for the inverted‐type polymer:fullerene solar cells). Finally, the PCE of the PBDB‐T:IT‐M solar cells could reach 12.2% by employing the P‐PI interlayers.

## 4 Experimental Section

*Materials and Solutions*: The detailed synthesis procedures for monomers (DNQT and DAQT) and precursor polymers (P‐PAA and C‐PAA) are given in the “Methods for Synthesis and Characterization” section in the Supporting Information. PBDB‐T (number‐average molecular weight = 18 kDa, polydispersity index = 2.5) and IT‐M were supplied from Solarmer Materials Inc. (China), while ITIC was purchased from Cal‐OS (USA). The PAA solutions were prepared by adding P‐PAA or C‐PAA to DMAc at a solid concentration of 3 mg mL^−1^. For the preparation of ZnO precursor solutions, zinc acetate dihydrate (100 mg) was dissolved in 2‐methoxyethanol (1 mL) together with ethanolamine (28 µL) and stirred on a hot plate at 60 °C for 4 h and then at 25 °C for 24 h. Solutions of PBDB‐T and nonfullerene acceptors (ITIC or IT‐M) for the BHJ layers were prepared by employing chlorobenzene (CB) and 1,8‐diiodooctane (DIO) (CB:DIO = 99:1 by volume) at a concentration of 20 mg mL^−1^ (PBDB‐T:nonfullerene acceptor = 1:1 by weight). The BHJ solutions were subjected to stirring at 50 °C before spin‐coating.

*Film and Device Fabrication*: Glass substrates coated with indium–tin oxide (ITO) layers (sheet resistance = ≈10 Ω cm^−2^) were subjected to the patterning process via photolithography/etching steps for making the discrete ITO electrodes. Acetone and isopropyl alcohol were used to clean the patterned ITO‐coated glasses, followed by the UV–ozone treatment for 20 min inside a UV–ozone cleaner (AC‐6, Ahtech LTS). Then, the ZnO precursor solutions were spun on the ITO–glass substrates to deposit the ZnO precursor layers, which were further annealed at 200 °C for 1 h in a typical laboratory environment. Next, the polyimide precursor layers (P‐PAA and C‐PAA) were spin‐coated on the ZnO layers, followed by thermal imidization at 200 °C for 90 min leading to corresponding PI layers (P‐PI and C‐PI). The thickness of the PI layers was ≈2 nm. All the film‐coated substrates were moved to a glove box charged with nitrogen gas. On top of the PI layers, the BHJ layers (PBDB‐T:ITIC and PBDB‐T:IT‐M) were deposited by spin‐coating at 2500 rpm. The BHJ layer (100 nm thick)–coated substrates were subjected to thermal annealing at 100 °C for 15 min inside the glove box, and then shifted into a thermal evaporation chamber inside an argon‐filled glove box. When the base pressure reached ≈1 × 10^−6^ Torr, the hole‐collecting buffer layer of molybdenum oxide (MoO_3_, ≈10 nm) was first deposited on the BHJ layers and then the silver electrode (Ag, ≈80 nm) was deposited on the MoO_3_ layer via the thermal evaporation process, which defined the active area of 0.05 cm^2^. The similar processes were applied for the fabrication of the inverted‐type polymer:fullerene solar cells (PTB7‐Th:PC_71_BM = 1:1.5 by weight, CB:DIO = 97:3 by volume, and active layer thickness ≈90 nm). The electron‐only devices (glass/ITO/ZnO/LiF/Al, glass/ITO/ZnO/P‐PI/LiF/Al, and glass/ITO/ZnO/C‐PI/LiF/Al) were fabricated by employing the same conditions for the ZnO and PI interlayers as for the solar cell fabrication, while lithium fluoride (LiF, ≈1 nm) and aluminum (Al, 90 nm) were sequentially deposited on the ZnO or PI interlayers inside the same vacuum chamber. All devices fabricated were stored in the same argon‐filled glove box before measurement.

*Measurements*: The film thickness was controlled by utilizing a surface profilometer (Alpha Step 200, KLA‐Tencor and DektakXT, Bruker), while the thickness of the PI interlayers was examined and calibrated with a UV–visible–NIR spectrometer (Lambda 750, PerkinElmer). The optical absorption spectra of film samples were measured using a UV–visible spectrometer (Optizen 2120, MECASYS) and a UV–visible–NIR spectrometer (Lambda 750, PerkinElmer), while the ionization potential of the PI films was measured using a photoelectron yield spectrometer (AC‐2, Hitachi High Tech). The FT‐IR spectra of the PAA and PI films were measured using an attenuated total reflection (ATR) FT‐IR spectrometer (Nicolet Continuum, Thermo Fisher Scientific). A Kelvin probe (KP) system (a 2 mm gold tip, APS01, KP Technology) was used to measure the work function of the ZnO films with and without the PI interlayers. The surface nanomorphology of samples was measured with an atomic force microscope (AFM, Nanoscope IIIa, Digital Instruments). The XPS spectra of the ZnO films with and without the PI interlayers were measured using an XPS spectrometer (ESCALAB 250Xi, Thermo Fisher Scientific). The impedance spectra (Nyquist plots) of solar cells were measured using an impedance spectrometer (VersaSTAT 4, Ametek Scientific Instruments). The solar cell performances, i.e., current density–voltage (*J*–*V*) characteristics, were measured using a home‐built measurement system with a solar simulator (class A, air mass 1.5 global, 92250A‐1000, Newport‐Oriel) and a source‐measure unit (2400, Keithley Instruments). The solar light intensity was adjusted to be 100 mW cm^−2^ (1 sun) by using a standard (calibrated) cell (BS‐520, Bunkoukeiki Co., Ltd) accredited by the Advanced Institute of Science and Technology (AIST, Japan). The EQE spectra were measured using a specialized measurement system equipped with a monochromator (CM110, Spectral Products) and a broadband light source (tungsten–halogen lamp, 150 W, ASBN‐W, Spectral Products). The short‐term stability test was carried out under continuous illumination with the simulated solar light (1 sun condition) at room temperature. All devices were safely mounted inside a nitrogen‐filled sample holder during the stability test.

## Conflict of Interest

The authors declare no conflict of interest.

## Supporting information

Supplementary

Supplementary

Supplementary
