# Supplementary material for: High‐Efficiency Polymer:Nonfullerene Solar Cells with Quaterthiophene‐Containing Polyimide Interlayers
Source: Adv Sci (Weinh). 2018 Jun 10;5(8):1800331. doi: 10.1002/advs.201800331 (PMC6097094; doi:10.1002/advs.201800331)
Supplement: Supplementary file 1 — Supplementary [file ADVS-5-1800331-s002.pdf]

Copyright WILEY-VCH Verlag GmbH & Co. KGaA, 69469 Weinheim, Germany, 2018.

ADVANCED SCIENCE
Open Access

Supporting Information

for *Adv. Sci.,* DOI: 10.1002/advs.201800331

High-Efficiency Polymer:Nonfullerene Solar Cells with Quaterthiophene-Containing Polyimide Interlayers

Euyoung Park, Jooyeok Seo, Hyemi Han, Hwajeong Kim, and Youngkyoo Kim**

Copyright WILEY-VCH Verlag GmbH & Co. KGaA, 69469 Weinheim, Germany, 2018.

Supporting Information

High Efficiency Polymer:Nonfullerene Solar Cells with Quaterthiophene-Containing Polyimide Interlayers

Euyoung Park[1#], *Jooyeok Seo*[1#], *Hyemi Han*[1#], *Hwajeong Kim*[1,2]* *and Youngkyoo Kim*[1]*

[1]Organic Nanoelectronics Laboratory and KNU Institute for Nanophotonics Applications (KINPA), Department of Chemical Engineering, School of Applied Chemical Engineering, Kyungpook National University, Daegu 41566, Republic of Korea
[2]Priority Research Center, Research Institute of Advanced Energy Technology, Kyungpook National University, Daegu 41566, Republic of Korea

[#]Equal Contribution
*Corresponding Authors: Prof. Y. Kim and Dr. H. Kim
Email) ykimm@knu.ac.kr
Tel) +82-53-950-5616, Fax) +82-53-950-6615

Methods for synthesis and characterization

***Materials for Synthesis*:** 2-Bromo-5-nitrothiophene (BNT, purity: 97%), tetrakis(triphenylphosphine)palladium(0) ($Pd(PPh_3)_4$, purity: 99%), zinc (<10μm, purity: 98%), and ammonium chloride (NH_4Cl, purity: >99.9%) were purchased from Sigma-Aldrich (USA). 5,5'-Bis(trimethylstannyl)-2.2'-bithiophene (BTSBT) was purchased from Solarmer Materials Inc. (China), while pyromellitic dianhydride (PMDA, purity: >98%) and cyclobutane-1,2,3,4-tetracarboxylic dianhydride (CTCDA, purity: >98%) were purchased from TCI (Japan). Several solvents including tetrahydrofuran (THF, purity: >99.5%), methyl alcohol (MeOH, purity: >99.8%), isopropyl alcohol (IPA, purity: 99.5%), and N,N-dimethylacetamide (DMAc, anhydrous, purity: 99.8%) were purchased from TCI (Japan), Junsei Chemical Co., Ltd. (Japan), Duksan (Korea), and Sigma-Aldrich (USA) and used without further purification.

***Synthesis of 5,5'''-dinitro-2,2':5',2'':5'',2'''-quaterthiophene (DNQT)*:** 5,5'''-dinitro-2,2':5',2'':5'',2'''-quaterthiophene (DNQT) was synthesized by the Stille coupling reaction between 2-bromo-5-nitrothiophene (BNT) and 5,5'-bis(trimethylstannyl)-2,2'-bithiophene (BTSBT). First, BTSBT (0.5 g), BNT (0.42 g), and $Pd(PPh_3)_4$ (6 mg) were added to a 100 ml three-necked flask in a glove box filled with nitrogen gas. The flask with tight sealing was flushed with argon gas for 10 min at room temperature. After adding THF (20 ml), the mixture solutions were stirred at room temperature for well dissolution, leading to a light yellow solution, and further stirred at 75 ^{o}C for 12 h. Then $Pd(PPh_3)_4$ (6 mg) was additionally injected to the mixture solutions, followed by reflux for further activation with stirring for another 12 h. To finish the reaction, the resulting dark brown solutions were cooled down slowly, followed by removing the THF solvent from the solutions by rotary evaporation under vacuum. The concentrated solutions were subjected to precipitation process in MeOH and the resulting solids were washed thoroughly with warm MeOH in order to remove the unreacted materials and/or impurities. The precipitates were obtained through repeated filtration steps using a filter paper (pore size: 1 μm) and dried in vacuum at 50 ^{o}C for 24 h. This process, finally, resulted in a black solid with the synthesis yield of ca. 88%. The final product was well dissolved in common polar aprotic solvents such as THF, DMF, DMAc, and N-methylpyrrolidone (NMP). Characterization results: (1) Measured mass (m/z) = 420.0 (calculated mass for $C_{16}H_8N_2O_4S_4$ = 419.94, see Figure S1a); (2) Elemental analysis: Found (%) = C: 46.8, H: 2.2, N: 5.9, S: 27.8, O: 14.1 (calculated for $C_{16}H_8N_2O_4S_4$ (%) = C: 45.7, H: 1.9, N: 6.6, S: 30.5, O: 15.2); (3) Major ^{1}H-NMR (DMF-d_7, 500 MHz): δ (ppm) = 7.5357~7.6790 (-CH, 4H, marked as 'a'), 7.6943~7.8037 (-CH, 2H, marked as 'b'), 8.1227~8.2344 (-CH, 2H, marked as 'c') (see Figure S2a); (4) Major ^{13}C-NMR (DMF-d_7, 500 MHz): δ (ppm) = 124.5 (-CH, 4C, marked as 'a'), 128.9253~129.0189 (-CH, 2C, marked as 'b'), 131.9350~132.2384 (4C, marked as 'd') (see Figure S2b).

***Synthesis of 5,5'''-diamino-2,2':5',2'':5'',2'''-quaterthiophene (DAQT)*:** The DNQT synthesized above was reduced to 5,5'''-diamino-2,2':5',2'':5'',2'''-quaterthiophene (DAQT) using a mixture solvent

of THF and deionized water (DIW). DNQT (0.3 g), NH_4Cl (0.23 g), and Zn (1.16 g) were added to a 100 ml three-necked round-bottom flask inside a nitrogen-filled glove box. After taking out, the flask was allowed to flushing with argon gas. Then, THF (20 ml) was added to the mixture and vigorously stirred at room temperature. Next, DIW (3 ml) was added by dropwise and deoxygenated sufficiently with argon gas for 20 min. The resulting dark brown solutions were refluxed at 80 ^{o}C for more than 24 h. The resulting dark orange solutions were naturally cooled down to room temperature and the diluted solutions were transferred to a larger (500 ml) flask. After removing all solvents via rotary evaporation, the product was completely dissolved in THF (250 ml) by stirring on a hot plate at 50 ^{o}C for approximately 12 h. Then Zn dust, which is insoluble in the THF solvent, was removed through the repeated centrifugation process at 3000 rpm for 15 min. The remaining THF solutions containing the product were subject to filtration with a filter paper (pore size: 1 μm), followed by further rotary evaporation process of the filtrates. The concentrated solutions were dropped into excess DIW (250 ml) upon stirring. The precipitated dark brown solids were obtained via filtration with a filter paper (pore size: 3 μm) and dried under vacuum at 70 ^{o}C (overnight). The reduction yield was ca. 80%. Similar to DNQT, DAQT was well dissolved in common polar aprotic solvents but the degree of solubility between both compounds was slightly different. Characterization results: (1) Measured mass (m/z) = 360.1 (calculated mass for $C_{16}H_{12}N_2S_4$ = 359.99, see Figure S1b); (2) Elemental analysis: Found (%) = C: 50.1, H: 3.2, N: 6.6, S: 31.8 (calculated for $C_{16}H_{12}N_2S_4$ (%) = C: 53.3, H: 3.3, N: 7.7, S: 35.5); (3) Major ^{1}H-NMR (DMF-d_7, 500 MHz): δ (ppm) = 5.8833~6.0395 (-CH, 2H, marked as 'c'), 6.5735~6.6745 (-CH, 2H, marked as 'b'), 6.8605~6.9147 (-NH_2, 4H, marked as 'd'), 7.1045~7.1242 (-CH, 4H, marked as 'a') (see Figure S3a); (4) Major ^{13}C-NMR (DMF-d_7, 500 MHz): δ (ppm) = 124.1283~125.4001 (-CH, 4C, marked as 'a'), 127.6 (-CH, 2C, marked as 'b'), 132.9030 (4C, marked as 'd'), 137.7884 (-CNH_2, 2C, marked as 'f') (see Figure S3b).

***Synthesis of PMDA-DAQT PAA (P-PAA)*:**

Poly(2-((5'''-methyl-[2,2':5',2'':5'',2'''-quaterthiophen]-5-yl)carbamoyl)-5-(methylcarbamoyl)terephthal-ic acid) (PMDA-DAQT PAA), namely P-PAA, was synthesized by polymerization of DAQT and pyromellitic dianhydride (PMDA). In a nitrogen-filled glove box, DAQT (50 mg) and PMDA (30.3 mg), separately, were added to the 50 ml round-bottom flasks sealed with a rubber septum (Precision Seal®). After taking out the flasks, they were flushed sufficiently with argon gas to further remove moisture and/or oxygen. Then, anhydrous DMAc was transferred to each flask using a hypodermic syringe equipped with a 1 ft long needle, which was prepared by repeated cleaning with argon gas. The solutions were stirred vigorously at room temperature, in which a little heat was applied to PMDA solution using a heat gun to dissolve completely. The concentration of each solution was 60 mg/ml. The completely dissolved PMDA solution was cooled down and added dropwise to the DAQT solution leading to the final concentration of 30 mg/ml. These solutions were stirred in an ice bath for 72 h. The resulting solutions exhibited gradual increase in viscosity. The final product, PMDA-DAQT PAA (P-PAA), was solidified by pouring the solutions into IPA upon stirring. The polymerization

yield was approximately 80%. The product (black solid) was stored in a refrigerator. The inherent viscosity of P-PAA was 2.87×10^{-2} dL/g in DMAc at a concentration of 0.7 g/dL at 35 ^{o}C.

***Synthesis of precursor polymer CTCDA-DAQT PAA (C-PAA)*:**

Poly(2-((5'''-methyl-[2,2':5',2'':5'',2'''-quaterthiophen]-5-yl)carbamoyl)-4-(methylcarbamoyl)cyclobutane-1,3-dicarboxylic acid) (CTCDA-DAQT PAA), C-PAA, was polymerized by reactions between DAQT and cyclobutane-1,2,3,4-tetracarboxylic dianhydride (CTCDA). DAQT (50 mg) and CTCDA (27.2 mg) were individually added to each 50 ml flask sealed with a rubber septum (Precision Seal®) inside a nitrogen-filled glove box. After taking out the flasks from the glove box, argon gas was flushed for 20 min to further remove oxygen and/or moisture. Then, anhydrous DMAc was added to each flask at a concentration of 60 mg/ml, followed by stirring at room temperature. The CTCDA solution was dropped to the DAQT solution and stirred at 0 ^{o}C for 72 h. After terminating the reaction, the final solutions were poured slowly into the 100 ml IPA bath upon stirring. The precipitated solids were recovered by filtration process and dried in a vacuum at room temperature for more than 24 h. The polymerization yield was ca. 70%. The inherent viscosity of C-PAA was 4.94×10^{-2} dL/g in DMAc at a concentration of 0.7 g/dL at 35 ^{o}C.

***Characterizations for the Synthesized Materials*:** The mass of monomers (DNQT and DAQT) was measured with a gas chromatography-mass spectrometer (GS-MS, 7890A-5975C GC/MSD, Agilent), while the elemental analysis was carried out using an elemental analyzer (EA, Flash 2000, Thermo Fisher Scientific). The chemical structure analysis was performed by employing a nuclear magnetic resonance spectrometer (^{1}H-NMR and ^{13}C-NMR, Avance III 500, Bruker), in which dinitro and diamino samples were measured in a solution of deuterated dimethylformamide (DMF-d_7) with tetramethylsilane (TMS) as an internal standard. The solution viscosity of poly(amic acid), P-PAA and C-PAA, was measured using a Cannon-Fenske type viscometer at 35 ^{o}C at a concentration of 0.7 g/dL. Then the intrinsic viscosity, [η], was determined from the relative viscosity (η_r) which was obtained by the different flow rates between the PAA solutions and solvent (DMAc). The intrinsic viscosity of the present PAAs was compared with that ([η] = 5.0×10^{-2} dL/g) of the commercial grade (solution) of poly(pyromellitic dianhydride-co-4,4'-oxydiamine) (PMDA-ODA PAA, product number: 593052, Sigma-Aldrich) at the same condition. The thermal transition temperature of PI samples, which were thermally imidized at either 200 ^{o}C for 90 min or 250 ^{o}C for 90 min, was measured using a differential scanning calorimeter (DSC, Q2000, TA Instruments) at a heating rate of 5 ^{o}C/min under a nitrogen atmosphere (note that actual DSC thermograms were obtained from the third run (heating rate: 5 ^{o}C/min) after the first (heating rate: 5 ^{o}C/min) and second (quenching) runs in order to remove possible thermal history of the PI samples). The thermal degradation characteristics of the samples were measured using a thermogravimetric analyzer (TGA, Q600, TA Instruments) at a heating rate of 5 ^{o}C/min.

Table S1. Summary of recent reports on the interlayers used for high efficiency organic solar cells with nonfullerene acceptors.

Author (Affiliation)	Device Structure	Interlayers	PCE (%)	Year	Ref.#
Y. Zang (Washington Univ., USA)	***Inverted Structure*** [ITO/ZnO/$PC_{61}BM$-SAM/PBDTT-F-TT:di-PBI/MoO_3/Ag]	$PC_{61}BM$-SAM (Small Molecule) Semiconducting Type	5.9	2014	15 (R1)
Y. Hwang (Washington Univ., USA)	***Inverted Structure*** [ITO/ZnO/PEI/PNDIS-HD:PBDTT-FTTE/MoO_3/Ag]	PEI (Polymer) Insulating Type	7.2	2015	18 (R2)
L. Reshma (VIT Univ., India)	***Inverted Structure*** [ITO/SnO_2/PEIE/P3HT:P(NDI2OD-T2)/MoO_3/Al]	PEIE (Polymer) Insulating Type	5.6	2017	20 (R3)
K. Zhang (Ocean Univ., China)	***Normal Structure*** [ITO/PFSs/PBDB-T:ITIC/PFN-Br/Al]	PFS (Polymer) Ionic Type	10.5	2017	21 (R4)
Z. Zhang (Beijing Normal Univ., China)	***Inverted Structure*** [ITO/ZnO/PFN-Br/PBDB-T:FTICs /MoO_3/Ag]	PFN-Br (Polymer) Ionic Type	11.1	2017	24 (R5)
This Work	***Inverted Structure*** [ITO/ZnO/PI/PBDB-T:ITIC/MoO_3/Ag]	P-PI (Polymer) Insulating Type	12.2	2018	-

#Note that references are cited in the manuscript but listed below again for easy comparison.

[R1] Y. Zang, C.-Z. Li, C.-C. Chueh, S. T. Williams, W. Jiang, Z.-H. Wang, J.-S. Yu, A. K.-Y. Jen, *Adv. Mater.* **2014**, *26*, 5708.

[R2] Y. Hwang, B. A. E. Courtright, A. S. Ferreira, S. H. Tolbert, S. A. H. Jenekhe, *Adv. Mater.* **2015**, *27*, 4578.

[R3] L. Reshma, K. Santhakumar, *Electrochim. Acta* **2017**, *250*, 267.

[R4] K. Zhang, X.-Y. Liu, B.-W. Xu, Y. Cui, M.-L. Sun, J.-H. Hou, *Chin. J. Polym. Sci.* **2014**, *35*, 219.

[R5] Z. Zhang, M. Li, Y. Liu, J. Zhang, S. Feng, X. Xu, J. Song, Z. Bo, *J. Mater. Chem. A* **2017**, *5*, 7776.

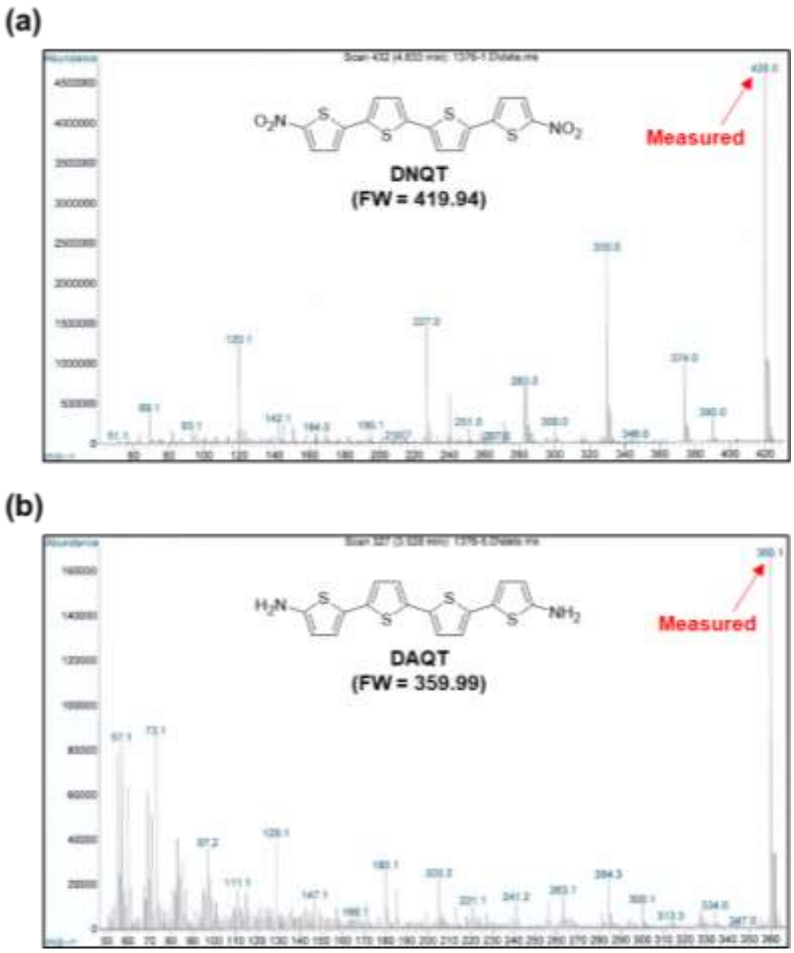

Figure S1. GC-MS spectra for the monomers synthesized in this work: (a) Dinitro compound (DNQT), (b) diamino compound (DAQT). The main peak of each spectrum, which is marked with a red arrow, delivered the charge-to-mass ratios (m/z = 420.0 for DNQT and 360.1 for DAQT) (inset: the calculated values for each compound). Note that small peaks can be assigned to the dissociation of (unstable) molecular ions generated during measurements.

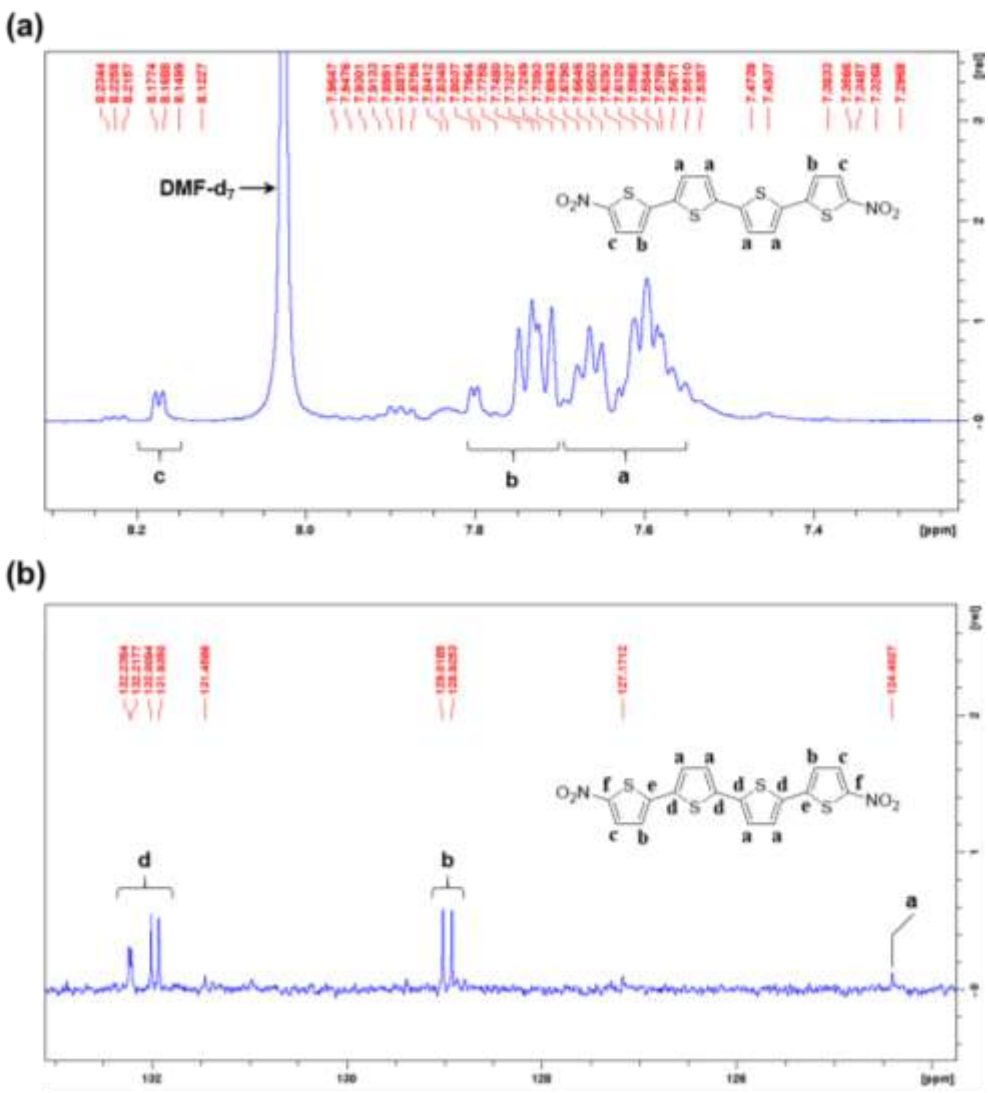

Figure S2. (a) ^{1}H-NMR and (b) ^{13}C-NMR spectra for the dinitro compound (DNQT) synthesized in this work (solvent: DMF-d_7). The detailed interpretation (assignment) of peaks is given in the method section.

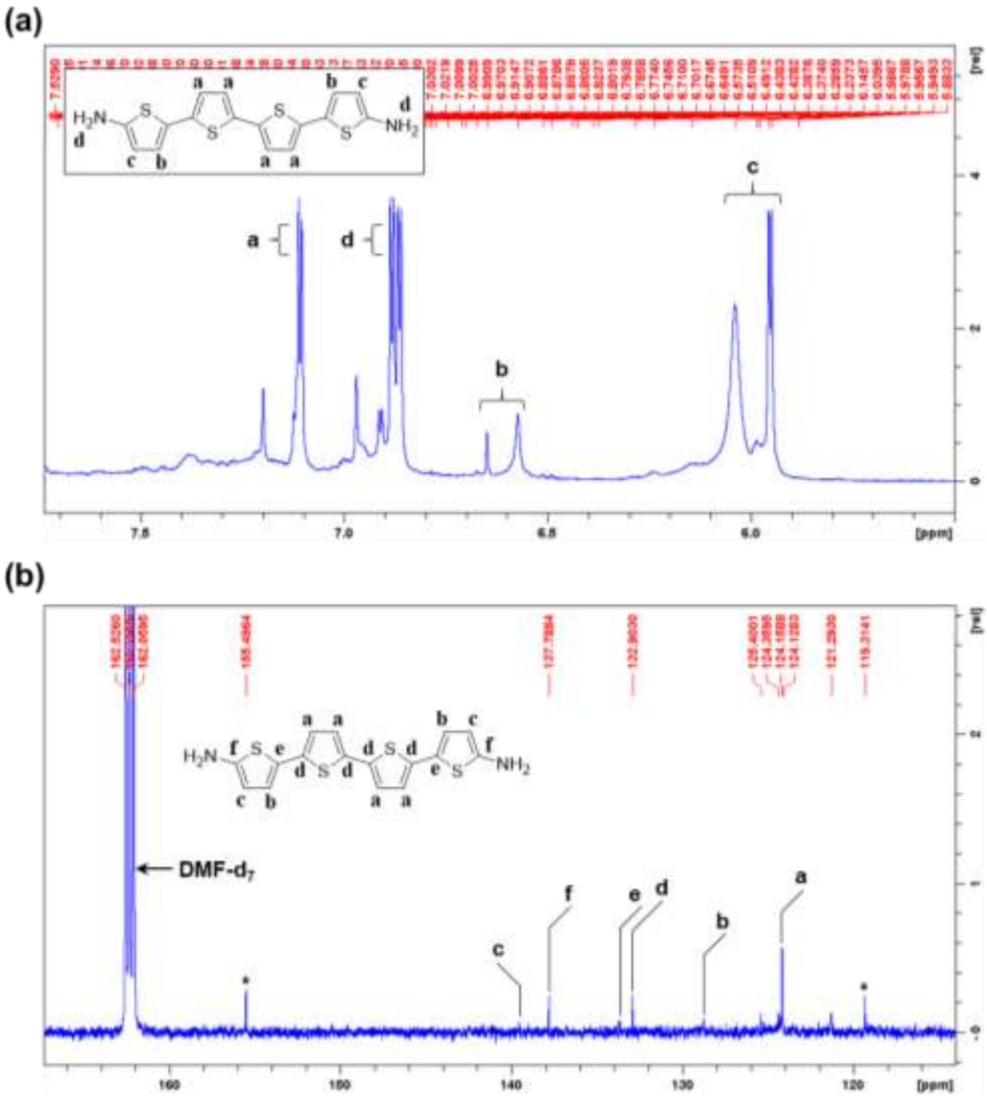

Figure S3. (a) ^{1}H-NMR and (b) ^{13}C-NMR spectra for the diamino compound (DAQT) synthesized in this work (solvent: DMF-d_7). The detailed interpretation (assignment) of peaks is given in the method section.

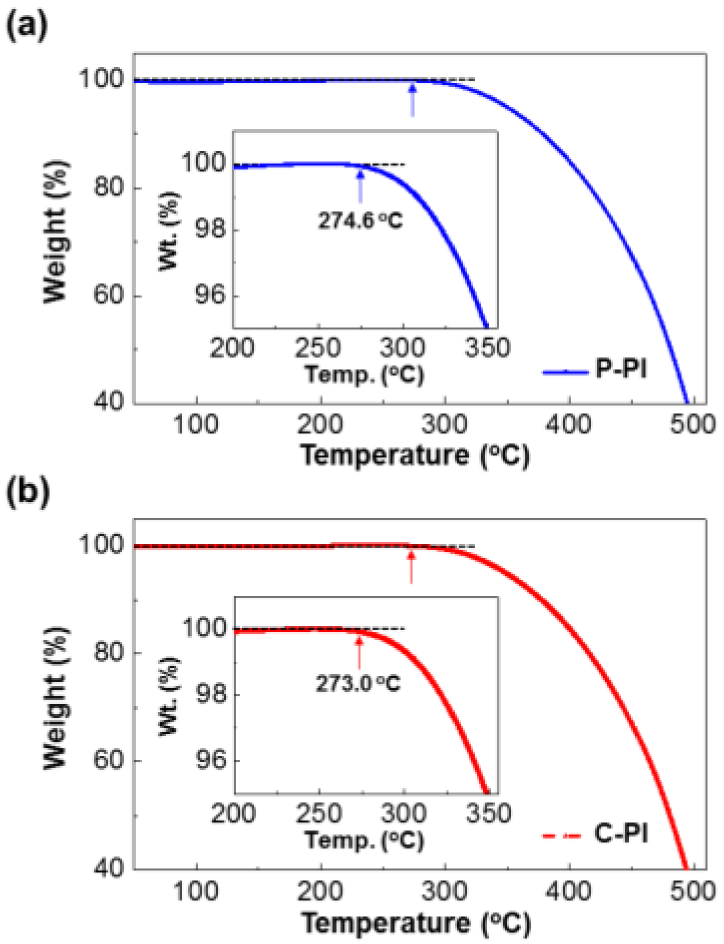

Figure S4. TGA thermograms for quarterthiophene-containing polyimides: (a) PMDA-based PI (P-PI) and (b) CTCDA-based PI (C-PI) (inset: enlarged part on the onset of weight loss). The thermal degradation temperature (onset) was measured ca. 274.6 °C (P-PI) and 273.0 °C (C-PI). Note that the PI samples used in this analysis were thermally imidized at 250 °C for 90 min.

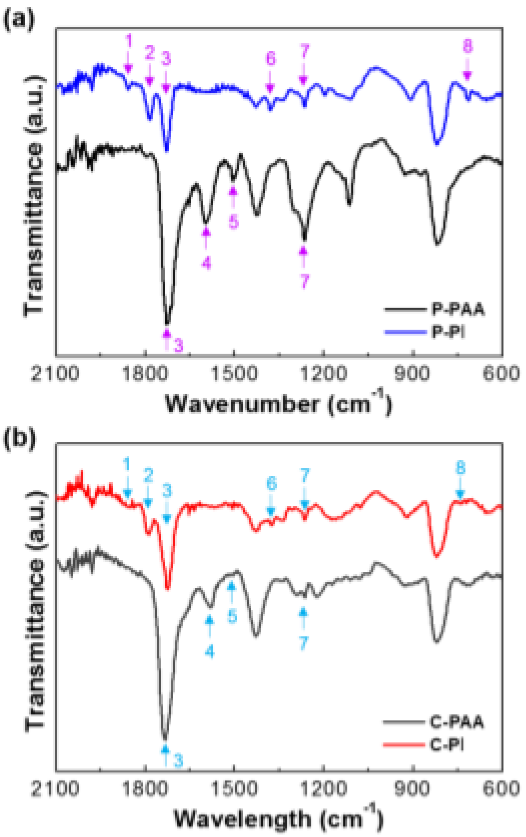

(a)

No.	Wavenumber (cm^{-1})	Functional Group	Vibration Characteristics
1	1856	Imide	C=O asymmetric stretching
2	1787	Imide	C=O symmetric stretching
3	1730	Carboxylic Acid	C=O stretching
4	1600	Amide	N-H bending
5	1506	Amide	C-N stretching
6	1380	Imide	C-N stretching
7	1265	Amide	C-N bending
8	716	Imide	C=O bending

(b)

No.	Wavenumber (cm^{-1})	Functional Group	Vibration Characteristics
1	1856	Imide	C=O asymmetric stretching
2	1790	Imide	C=O symmetric stretching
3	1723	Carboxylic Acid	C=O stretching
4	1560	Amide	N-H bending
5	1506	Amide	C-N stretching
6	1376	Imide	C-N stretching
7	1263	Amide	C-N bending
8	740	Imide	C=O bending

Figure S5. FT-IR spectra of quarterthiophene-containing PAA and PI films (thermal imidization at 200 °C for 90 min): (a) P-PAA and P-PI, (b) C-PAA and C-PI. The numbers indicating the characteristic peaks are summarized in the table (right) for each case.

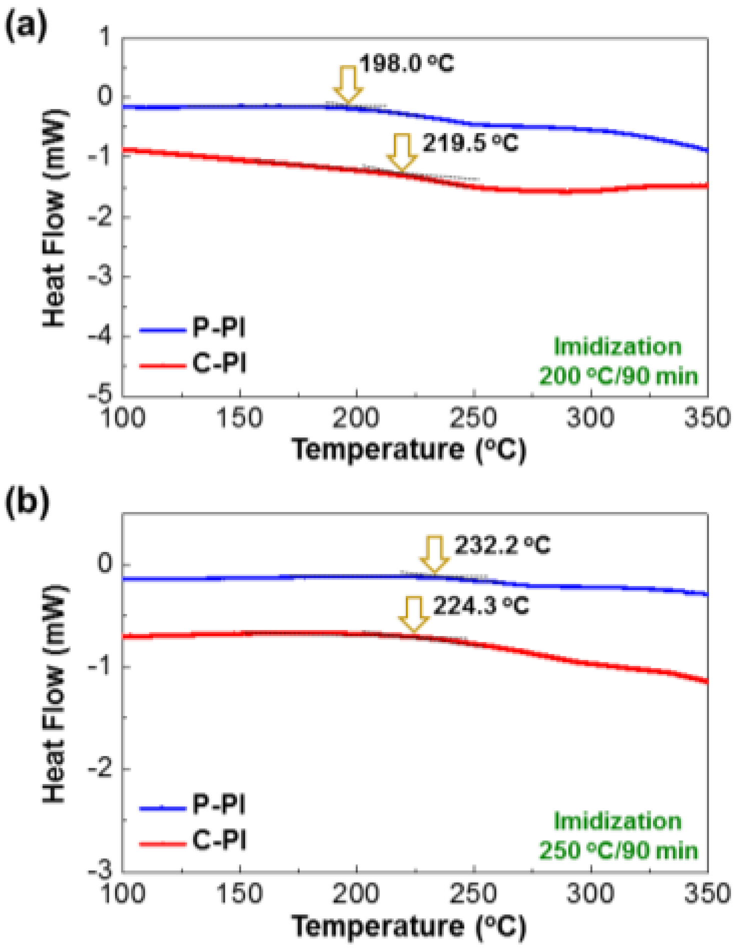

Figure S6. DSC thermograms for quarterthiophene-containing PIs imidized at different temperatures: (a) 200 °C for 90 min, (b) 250 °C for 90 min. The scan rate was 5 °C/min. The glass transition temperature (Tg) (onset) was measured >198 °C in the case of the samples imidized at 200 °C, while it was higher than 220 °C in the case of the samples imidized at 250 °C. Note that the weak and broad heat flow change at around Tg can be attributed to the nature of rigid polymers as reported in various literatures including *Chem. Mater.* 16, 5051 (2004), *Macromol. Res.* 18, 120 (2010), *Prog. Org. Coat.* 99, 103 (2016).

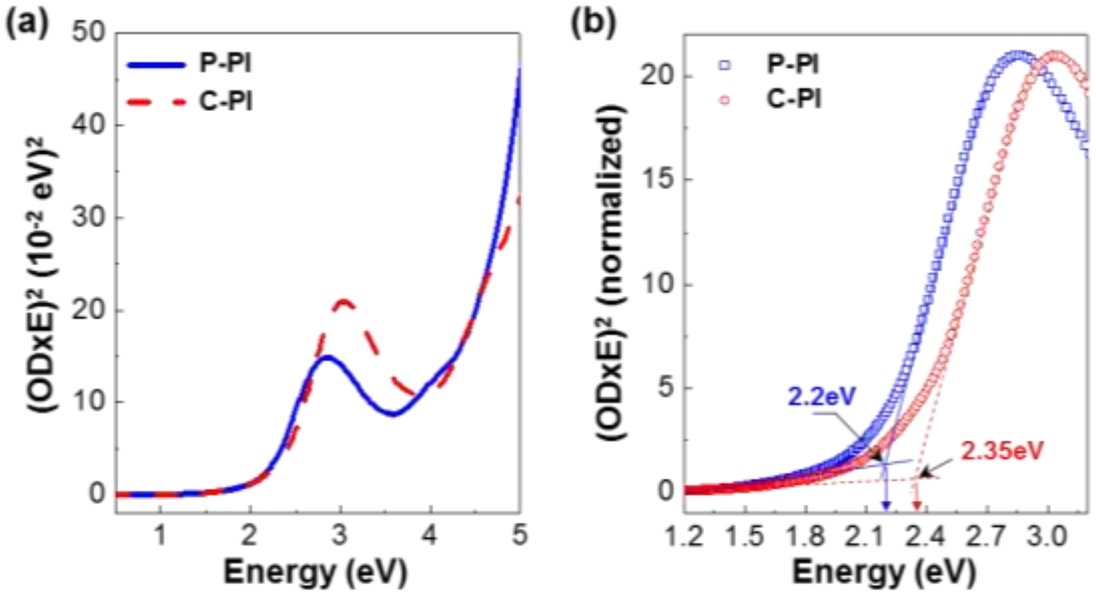

Figure S7. (a) Tauc plots for quarterthiophene-containing PIs: (a) full range, (b) band edge region (normalized). OD and E denote optical density and photon energy, respectively.

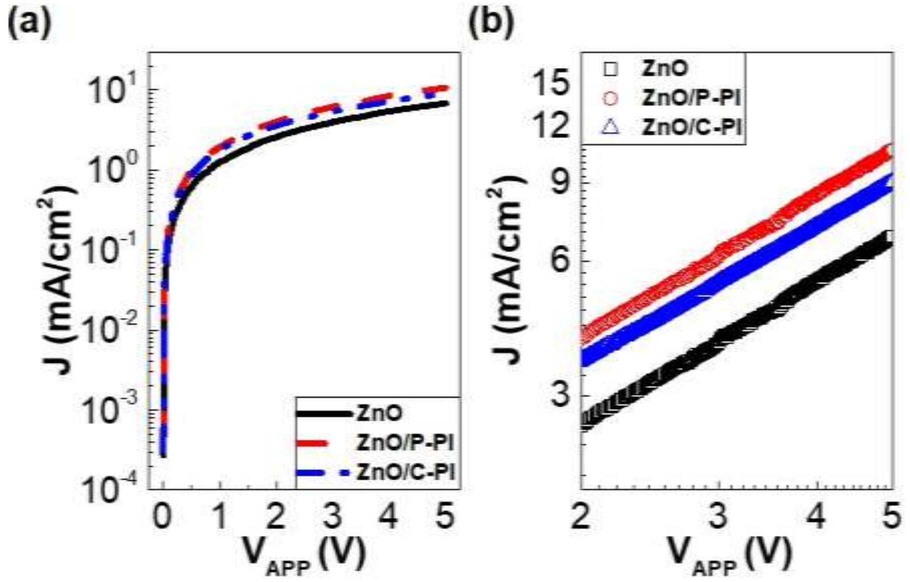

Figure S8. (a) Current density (J) as a function of effective voltage (V_{Eff}) for three different electron-only devices on a semi-logarithmic scale (glass/ITO/ZnO/LiF/Al, glass/ITO/ZnO/P-PI/LiF/Al, and glass/ITO/ZnO/C-PI/LiF/Al): V_{Eff} was obtained by subtracting the built-in voltage from the applied voltage (V_{APP}). (b) J-V_{Eff} curves on a double logarithmic scale in the voltage range of space-charge-limited current (SCLC) regime. The electron mobility was 2.5×10^{-6} $cm^2/V\cdot s$, 5.6×10^{-6} $cm^2/V\cdot s$, and 3.8×10^{-6} $cm^2/V\cdot s$ for ZnO, ZnO/P-PI, and ZnO/C-PI, respectively. The related literatures: J. Zhao et al. *Nat. Energy* 15027 (2016), H. Han et al. *J. Mater. Chem. A* DOI: 10.1039/C8TA00147B, S. Li et al. *Adv. Mater.* 29, 1704051 (2017), S. Xie et al. *Adv. Funct. Mater.* 28, 1705659 (2018).

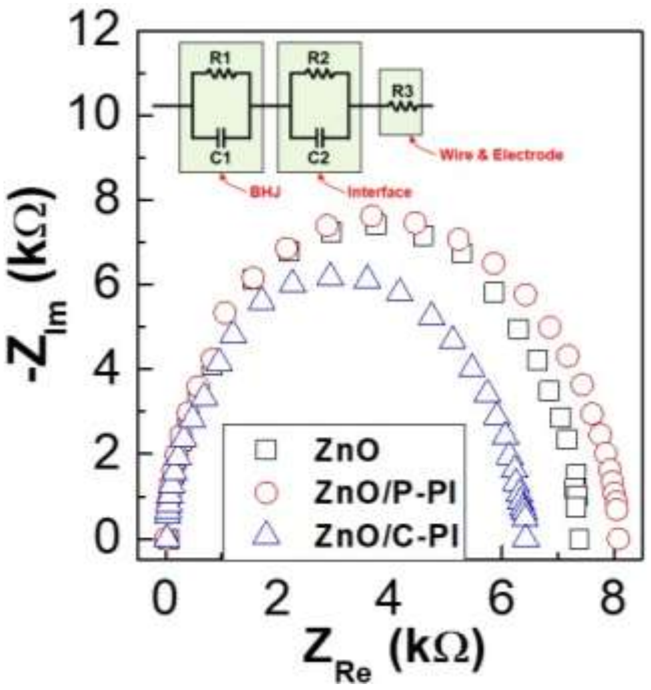

Figure S9. Nyquist plots for the inverted-type PBDB-T:ITIC solar cells with and without the PI interlayers at open-circuit condition in the frequency range between 1 Hz and 0.1 MHz. Z_{Re} and Z_{Im} denote real and imaginary impedances, respectively. The inset diagram shows an equivalent circuit diagram for the calculation of resistance and capacitance. The interfacial resistance (R2) was extracted to 159.6 Ω, 143.1 Ω, and 167.2 Ω for ZnO, ZnO/P-PI, and ZnO/C-PI, respectively. The related literatures: Ecker et al. *J. Phys. Chem. C* 116, 16333 (2012); Kuwabara et al. *ACS Appl. Mater. Interfaces* 1, 2107 (2009).

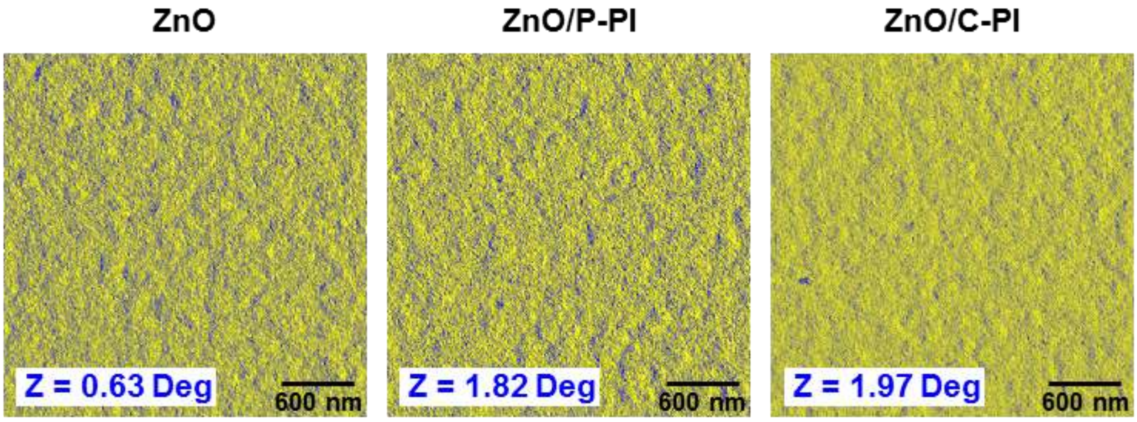

Figure S10. Phase-mode AFM images for the ZnO (left), ZnO/P-PI (middle), and ZnO/C-PI (right) films coated on ITO-glass substrates. The scan size of the AFM images was 3 μm × 3 μm. The phase angle (Z) is given on each image.

(a)
(b)

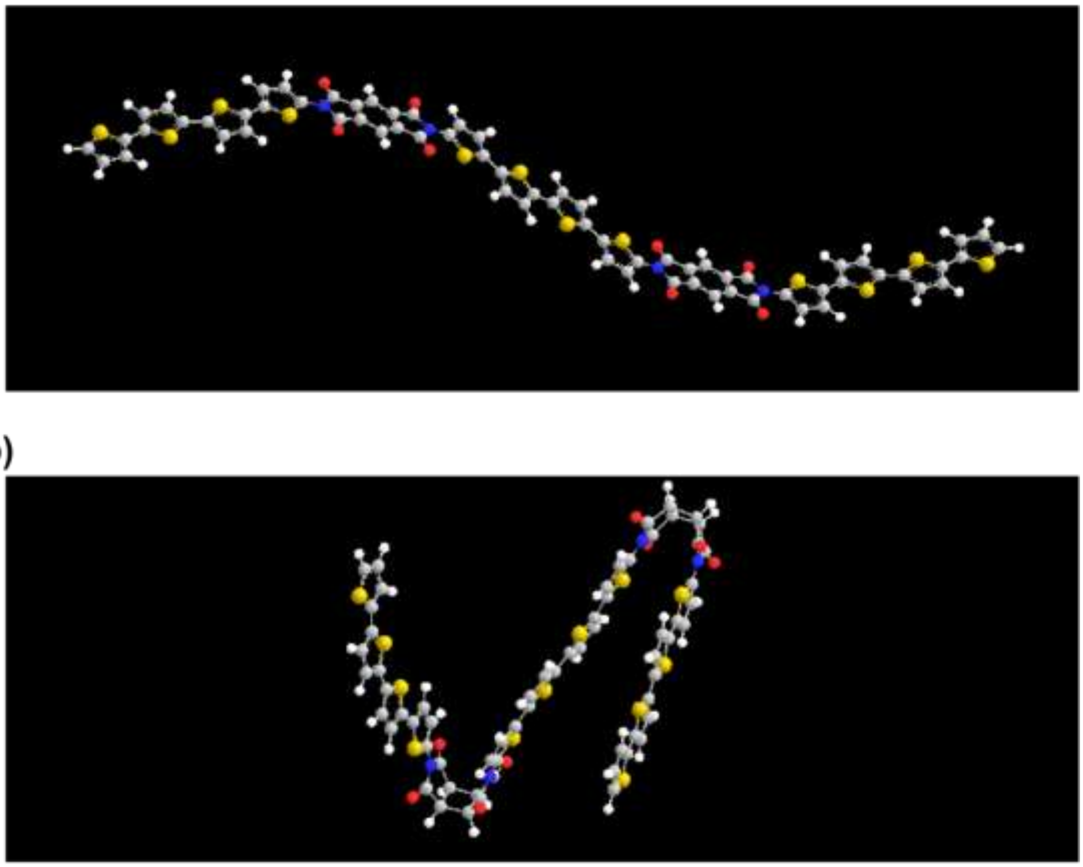

Figure S11. Energy minimized molecular structures for the three repeating units of quarterthiophene-containing polyimides: (a) PMDA-based PI (P-PI), (b) CTCDA-based PI (C-PI). See video clip for 3D structures (filename: P-PI_S11a.avi, C-PI_S11b.avi).

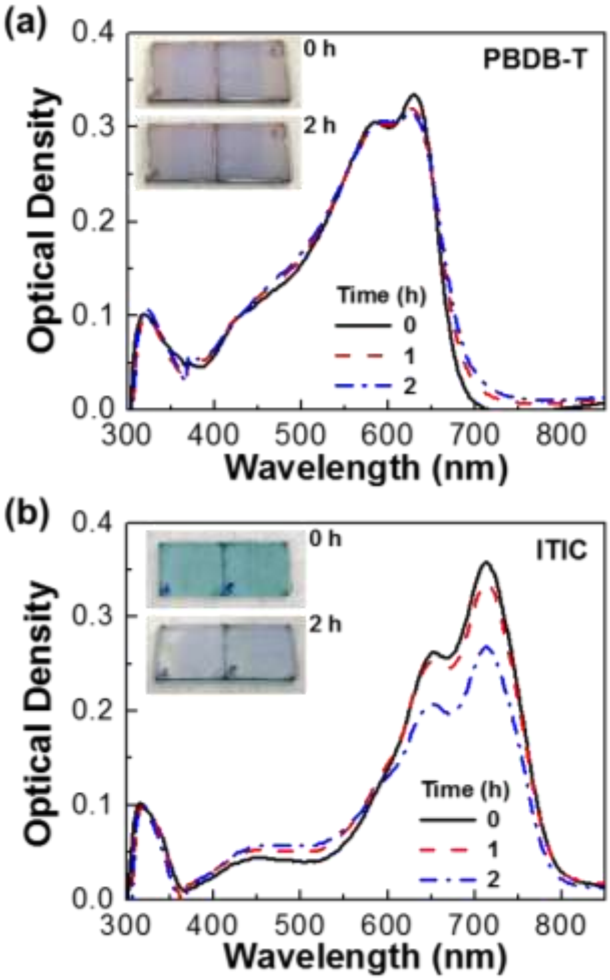

Figure S12. Optical absorption spectra for the pristine films (a: PBDB-T, b: ITIC) coated on ITO-glass substrates according to the exposure time under continuous illumination of a simulated solar light (air mass 1.5G, 100 mW/cm^2). The inset photographs show the color change of the pristine films before (0 h) and after illumination (2 h).

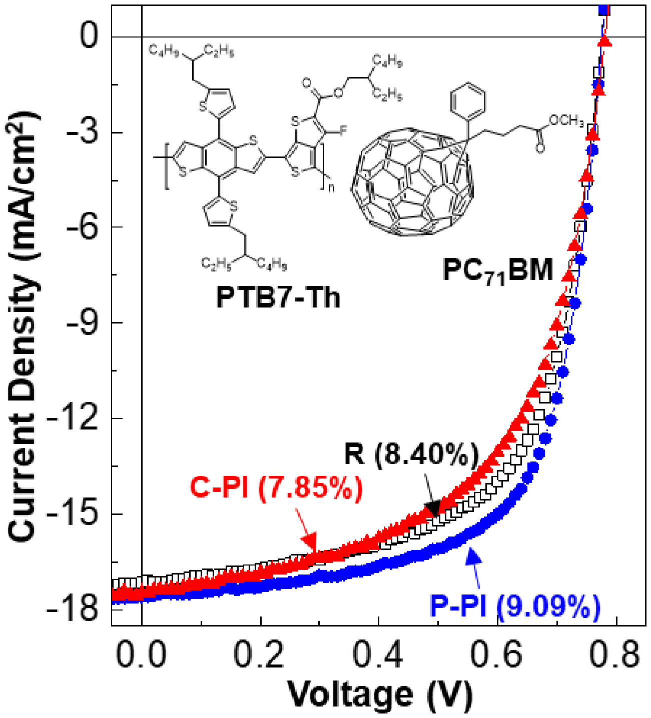

Figure S13. Light (air mass 1.5G, 100 mW/cm^2) J-V curves for the inverted-type PTB7-Th:PC$_{71}$BM solar cells without (**R**) and with the PI interlayers (**P-PI** and **C-PI**). **R**: J_{SC} = 17.13 mA/cm^2, V_{OC} = 0.77 V, FF = 63.7 %, PCE = 8.4 %; **P-PI**: J_{SC} = 17.57 mA/cm^2, V_{OC} = 0.78 V, FF = 67.1 %, PCE = 9.09 %; **C-PI**: J_{SC} = 17.44 mA/cm^2, V_{OC} = 0.77 V, FF = 57.7 %, PCE = 7.85 %.
